# HLA alleles, especially amino-acid signatures of HLA-DPB1, might contribute to the molecular pathogenesis of early-onset autoimmune thyroid disease

**DOI:** 10.1371/journal.pone.0216941

**Published:** 2019-05-15

**Authors:** Dong-Hwan Shin, In-Cheol Baek, Hyung Jae Kim, Eun-Jeong Choi, Moonbae Ahn, Min Ho Jung, Byung-Kyu Suh, Won Kyoung Cho, Tai-Gyu Kim

**Affiliations:** 1 Department of Microbiology, College of Medicine, The Catholic University of Korea, Seoul, Korea; 2 Department of Biomedicine & Health Sciences, College of Medicine, The Catholic University of Korea, Seoul, Korea; 3 Hematopoietic Stem Cell Bank, College of Medicine, The Catholic University of Korea, Seoul, Korea; 4 Department of Pediatrics, College of Medicine, The Catholic University of Korea, Seoul, Korea; Istituto di Ricovero e Cura a Carattere Scientifico Centro di Riferimento Oncologico della Basilicata, ITALY

## Abstract

The major histocompatibility complex region has been suggested to play an important role in the development of autoimmune thyroid disease (AITD). In this study, we investigated the associations of human leukocyte antigen (HLA) alleles and amino acid variants of HLA with early-onset AITD. HLA class I and class II genes were analyzed in 116 Korean children with AITDs (Graves’ disease [GD]: 71, Hashimoto’s disease [HD]: 45) and 142 healthy controls. HLA-B*46:01 (OR = 3.96, *Pc* = 0.008), -C*01:02 (OR = 2.51 *Pc* = 0.04), -DPB1*02:02 (OR = 3.99, *Pc* = 0.04), and -DPB1*05:01 (OR = 4.6, *Pc* = 0.003) were significantly associated with GD, and HLA-A*02:07 (OR = 4.68, *Pc* = 0.045) and -DPB1*02:02 (OR = 6.57, *Pc* = 0.0001) with HD. The frequency of HLA-DPB1*05:01 was significantly higher in GD patients than in HD patients (*Pc* = 0.0005). Furthermore, differences were found between patients with Thyroid associated ophthalmopathy (TAO) and those without TAO in the distribution of HLA-B*54:01 (8.6% vs. 30.6%, *P* = 0.04) and -C*03:03 (37.1% vs. 11.1%, *P* = 0.02). In the analysis of amino acid variants of HLA molecules, both Leu35 (OR = 23.38, *P* = 0.0002) and Glu55 (OR = 23.38, *P* = 0.0002) of HLA-DPB1 were strongly associated with GD and showed different distributions between GD and HD (*P* = 0.001). Our results suggest that HLA alleles, especially amino-acid signatures of the HLA-DP β chain, might contribute to the molecular pathogenesis of early-onset AITD.

## Introduction

Autoimmune thyroid diseases (AITD) is composed of two main clinical entities, Graves’ disease (GD) and Hashimoto’s disease (HD), and is characterized by infiltration of the thyroid by T cells and B cells and the production of antibodies specific for thyroid antigens [1]. Among immune-related genes, human leukocyte antigen (*HLA*), cytotoxic T lymphocyte-associated factor 4 (*CTLA-4*), CD40, and protein tyrosine phosphatase-22(*PTPN22*) have been found to be associated with susceptibility to AITD. In particular, because the major histocompatibility complex region is highly polymorphic and relevant to many immune response genes, HLA is the most prominent candidate genetic factor for several autoimmune diseases, including AITD [2].

Previous genetic association studies with AITD have shown some ethnic differences [3]. In Caucasians, associations of HLA-DQA1*0501 and -DQB1*0301, -DRB1*03, and -DRB1*08 with GD and of HLA-DRB1*03, -DRB1*04, -DRB1*08, -DQA1*03011/12, -DQA1*0401, and -DQB1*0301/4 with HD were reported [4, 5]. In the Japanese population, HLA-B*35:01, -B*46:01,-DRB1*14:03, -DQB1*0604, and -DPB1*05:01 were found to be positively associated with GD, and HLA-A*02:07 and -DRB4 were found to be positively associated with HD [6]. In the Chinese population, HLA-A*11:01/02, -B*46:01, -DRB1*15:01, -DRB1*16:02, -DQB1*05:02, and -DPB1*0501 were found to be positively associated with GD [7]. In the Korean population, HLA-DRB1*030101, -DRB1*080201,-DRB1*0803, -DRB1*140301, and-DRB1*1602 were found to be positively associated with GD [8, 9].

Genetic susceptibility may be a greater concern for early-onset AITD than for late-onset AITD. A previous study by our research group identified HLA-B*46, -DRB1*08, and -Cw*01 as disease-susceptibility alleles for early-onset AITD, with greater statistical significance than for late-onset AITD [10]. Furthermore, different allelic associations of HLA have been reported between early-onset GD and late-onset GD in Japanese studies. In particular, HLA-DPB1*0501 is strongly associated with early-onset GD in Japanese [11]. These findings jointly suggest that early-onset AITD may be more strongly influenced by genetic factors than late-onset AITD.

Recently, disease association studies on HLA have shifted focus from the identification of HLA alleles associated with disease susceptibility to the molecular structure of HLA [12]. Previously reported disease risk associated amino acids have been frequently located in the peptide-binding grooves or functional pockets of HLA molecules, suggesting their functional contributions to antigen-presentation ability or protein stability [13]. The amino acid polymorphisms of HLA class I and class II genes independently contribute to disease risk, as has been identified by applying HLA imputation to genome-wide association study data for GD [14]. In this study, we sought to identify associations of HLA class I and II alleles and amino acid variants with early-onset AITD, and further investigated whether there were differences in HLA associations among the constituent diseases of AITD.

## Materials and methods

### Subjects

This study was conducted on 116 patients diagnosed with AITD (GD: 71 and HD: 45) who were treated at the pediatric endocrine clinic of Seoul St. Mary’s Hospital between March 2009 and February 2018. Fifty-five of these patients were also included in a previous study by our research group [15]. Only pediatric AITD patients without other clinically-identified diseases were included in the case group. The age of patients at enrollment in the study was 13.8 ± 3.4 years, and the age at diagnosis of AITD was 11.4 ± 3.1 years (Table 1).

**Table 1:** Characteristic of 116 AITD patients.

Characteristic	
Sex (F/M)	95/21
Age (years) at diagnosis	11.4 ± 3.1
5~10	48(41.4%)
11~13	40(34.5%)
14~17	25(21.6%)
> 17	3(2.6%)
Age (years) at enrollment	13.8 ± 3.4
HD/GD	45 / 71
HD condition at diagnosis	
Euthyroid state	9(20.0%)
Subclinical hypothyroid state	16(35.6%)
Overt hypothyroid state	15(33.3%)
Hyperthyroid state	5(11.1%)
Class of TAO	
0~1 No sign~ only sign	36(50.7%)
2 soft tissue involvement	8(11.3%)
3 Proptosis	23(32.4%)
4 Extraocular muscle involvement	3(4.2%)
5 Corneal involvement	1(1.4%)
6 Sight loss	0(0.0%)

AITD, autoimmune thyroid diseases; HD, Hashimoto’s disease; GD, Graves’ disease; TAO = 32, thyroid associated ophthalmopathy

The control group included 142 genetically unrelated healthy Korean adults (age: 30s; 70 female and 72 male) without a history of AITD. The control group mainly comprised students and staff from the Medical College of the Catholic University of Korea and hematopoietic stem cell transplantation (HSCT) center.

### Ethics statement

All subjects provided informed consent to participate in a genetic study. Also, written informed consent was obtained from each children participant and their parents or guardians. The Institutional Review Board (IRB) of the Catholic University of Korea approved our study. (IRB Number: KC09FISI0042, MC13SISI0126, VC17TESI0129)

### Diagnosis of clinical features

HD was diagnosed when at least three of Fisher’s criteria [16] were met: (1) goiter, (2) diffuse goiter and decreased uptake on a thyroid scan, (3) the presence of circulating thyroglobulin and/or thyroid peroxidase antibody (TPOAb), and (4) hormonal evidence of hypothyroidism. The GD diagnosis was based on the confirmation of clinical symptoms and the biochemical confirmation of hyperthyroidism, including observations of goiter, positive TSH-receptor antibodies, and elevated thyroid hormone levels. Patients who had other autoimmune diseases, hematologic diseases, and endocrine diseases were excluded. Thyroid-associated ophthalmopathy (TAO) was diagnosed based on presence of typical clinical features and classified according to the system recommended by the American Thyroid Association Committee. Patients with no symptoms or only the lid lag sign were included in the without-TAO group [17]. Patients with soft tissue changes, proptosis, extraocular muscle dysfunction, or the latter two symptoms were considered to have an eye disease [18].

### DNA extraction

Genomic DNA was extracted from 4 mL of peripheral blood mixed with ethylenediaminetetraacetic acid using TIANamp Genomic DNA Extraction Kits (Tiangen Biotech Corporation, Beijing, China), according to the manufacturer’s instructions. The concentration of the DNA solution was adjusted to 100 ng/μL, and the solution was stored at -20°C. It was used as a polymerase chain reaction (PCR) template for genotyping.

### HLA genotyping

For screening of HLA-A, -B, -C, -DRB1, -DQB1, and -DPB1 alleles, genotyping of HLA-A, -B, -C, -DRB1, -DQB1, and -DPB1 was performed using PCR-sequence-based typing (PCR-SBT) methods. The majority of HLA class I alleles can be distinguished by their exon 2 and 3 sequences, and for class II alleles, exon 2 is generally sufficient. Accordingly, exons 2 and 3 of HLA-A, -B, and -C and exon2 of HLA-DRB1, -DQB1, and -DPB1 were amplified by PCR. Briefly, exons 2 and 3 of HLA-A, -B, and -C were amplified as recommended by the 13th International Histocompatibility Workshop [19], and exon 2 of HLA-DRB1 and -DQB1 was amplified by nested-PCR in two steps: (1) primer pairs (DRBF/DRBR and DQBF/DQBR) and (2) specific pairs of primers [20, 21]. For exon 2 of HLA-DPB1, amplification was performed using a single primer pair (DPBF / DPBR) [22]. PCR amplification was performed using a GeneAmp PCR system 9700 (Applied Biosystems, Foster City, CA, USA). The PCR products were purified with ABI PRISM BigDye Terminator Ready Reaction Kit (Applied Biosystems, Foster City, CA, USA) using an ABI 377 DNA sequencer (Applied Biosystems, Foster City, CA, USA), according to the manufacturer’s instructions. The raw sequencing data were analyzed using ABI Factura software and the ABI Sequence Navigator Program (Applied Biosystems, Foster City, CA, USA). In total, 18 alleles for HLA-A, 38 alleles for HLA-B, 21 alleles for HLA-C, 29 alleles for HLA-DRB1, 15 alleles for HLA-DQB1, and 13 alleles for HLA-DPB1 were detected, respectively.

### Analysis of amino acid variants in HLA class I/II

Amino acid sequences for HLA-A, -B, -C, -DRB1, -DQB1, and -DPB1 with a resolution of 4 digits were obtained from the ImMunoGeneTics references. Analysis of variant amino acids was performed across all the HLA-A, -B, -C, -DRB1, -DQB1, and -DPB1 alleles that were present in the genotyping results.

### Statistical analysis

In this study, the allele-carrying frequency was calculated by dividing the number of patients carrying the allele by the total number of group members. The alleles of each individual were coded with tested alleles in a two-allele format (at any HLA loci, there are multiple alleles). The allele-carrying frequencies of HLA were compared with the chi-square test or the Fisher exact probability test. Firth logistic regression with a dominant genetic model (i.e., carrying at least one copy of the allele is all that matters) was used to estimate the crude and sex-adjusted odds ratios (ORs) and 95% confidence intervals (CIs). We estimated haplotype frequencies using *haplo*.*em*, an implementation of an expectation-maximization (EM) algorithm included in the R package *haplo*.*stats*, which computes maximum likelihood estimates of haplotype probabilities from unphased genotypes measured on unrelated individuals. A corrected *P*-value (*Pc*) based on the Bonferroni correction was used for multiple test correction. *Pc* values < 0.05 were considered to indicate statistical significance. Statistical analysis was conducted using SAS version 9.4 (SAS Institute, Cary, NC, United States) and R version 3.5.2 (The R Foundation for Statistical Computing, Vienna, Austria). Hardy-Weinberg equilibrium in the controls and cases was analyzed for each single-nucleotide polymorphism (SNP) using the SNPStats website (http://bioinfo.iconcologia.net/snpstats/start.htm)(P > 0.05).

## Results

### Associations of HLA class I and class II alleles with AITD

In the analysis of associations of HLA class I (A, B, and C) and class II (DRB1, DQB1, and DPB1) alleles with GD, significant positive associations were found for HLA-A*02:07, -B*46:01 (OR = 3.96, *Pc* = 0.008), -C*01:02 (OR = 2.51, *Pc* = 0.04), -DRB1*08:03, -DRB1*14:03, -DPB1*02:02 (OR = 3.99, *Pc* = 0.04), and -DPB1*05:01 (OR = 4.6, *Pc* = 0.003) (Table 2). Significant positive associations with HD were found for HLA-A*02:07 (OR = 4.68, *Pc* = 0.045) and -DPB1*02:02 (OR = 6.57, *Pc* = 0.001). In addition to the above alleles, HLA-B*44:03, -C*06:02, -DRB1*01:01, -DQB1*02:02, and -DQB1*0501 (OR = 0.22, *Pc* = 0.03) were negatively associated with only AITD (S1 Table). Only HLA-DPB1*05:01 showed a stronger relationship with GD (*Pc* = 0.0005) than with HD. Among the HLA class I and II genes, HLA-A and HLA-DPB1 were most strongly associated with AITD. In the sex-adjusted analysis, HLA-A*33:03 showed a significant association with GD, and -DRB1*07:01 and -DRB1*14:03 did so for AITD; however, HLA-B*4403 and -DRB1*01:01 showed no associations with AITD patients (data of the sex-adjusted analysis not presented due to the absence of significant differences).

**Table 2:** Allele-carrying frequencies of HLA-A, -B, -C, -DRB1, -DQB1 and -DPB1 alleles significantly associated with GD or HD in Korean children with AITD (*P* < 0.01).

		Controls		AITD		GD		control vs GD		HD		control vs HD		GD vs HD
Locus	Allele	n = 142 (%)		n = 116 (%)		n = 71 (%)		OR (95% CI)	p value	n = 45 (%)		OR (95% CI)	p value	p value
A	02:07	8	(5.6)	24	(20.7)	14	(19.7)	3.99 (1.59–10.01)	0.003	10	(22.2)^f^	4.68 (1.72–12.73)	0.003	
B	46:01	16	(11.3)	35	(30.2)	24	(33.8)^a^	3.96 (1.94–8.09)	0.0002	11	(24.4)	2.56 (1.09–6.01)		
C	01:02	48	(33.8)	63	(54.3)	40	(56.3)^b^	2.51 (1.40–4.49)	0.002	23	(51.1)	2.04 (1.03–4.01)		
DRB1	08:03	20	(14.1)	35	(30.2)	22	(31.0)	2.72 (1.36–5.42)	0.005	13	(28.9)	2.48 (1.12–5.51)		
DRB1	14:03	2	(1.4)	9	(7.8)	8	(11.3)	7.52 (1.66–33.98)	0.009	1	(2.2)	1.90 (0.18–20.10)		
DPB1	02:02	8	(5.6)	27	(23.3)	14	(19.7)^c^	3.99 (1.59–10.01)	0.003	13	(28.9)^g^	6.57 (2.52–17.14)	0.0001	
DPB1	05:01	88	(62.0)	86	(74.1)	63	(88.7)^d^^,^^e^	4.60 (2.07–10.21)	0.0002	23	(51.1)	0.64 (0.33–1.26)		3.6E-05

AITD, autoimmune diseases; GD, Graves' disease; HD, Hashimoto's disease; OR, Odds ratio; CI, Confidence intervals. Control vs. GD

^a^*Pc* = 0.008

^b^*Pc* = 0.04

^c^*Pc* = 0.04

^d^*Pc* = 0.003. Control vs. HD

^f^*Pc* = 0.045

^g^*Pc* = 0.001.GD vs. HD

^e^*Pc* = 0.0005

### Associations of HLA class I and class II alleles with TAO or with non-TAO

**Fig 1:**
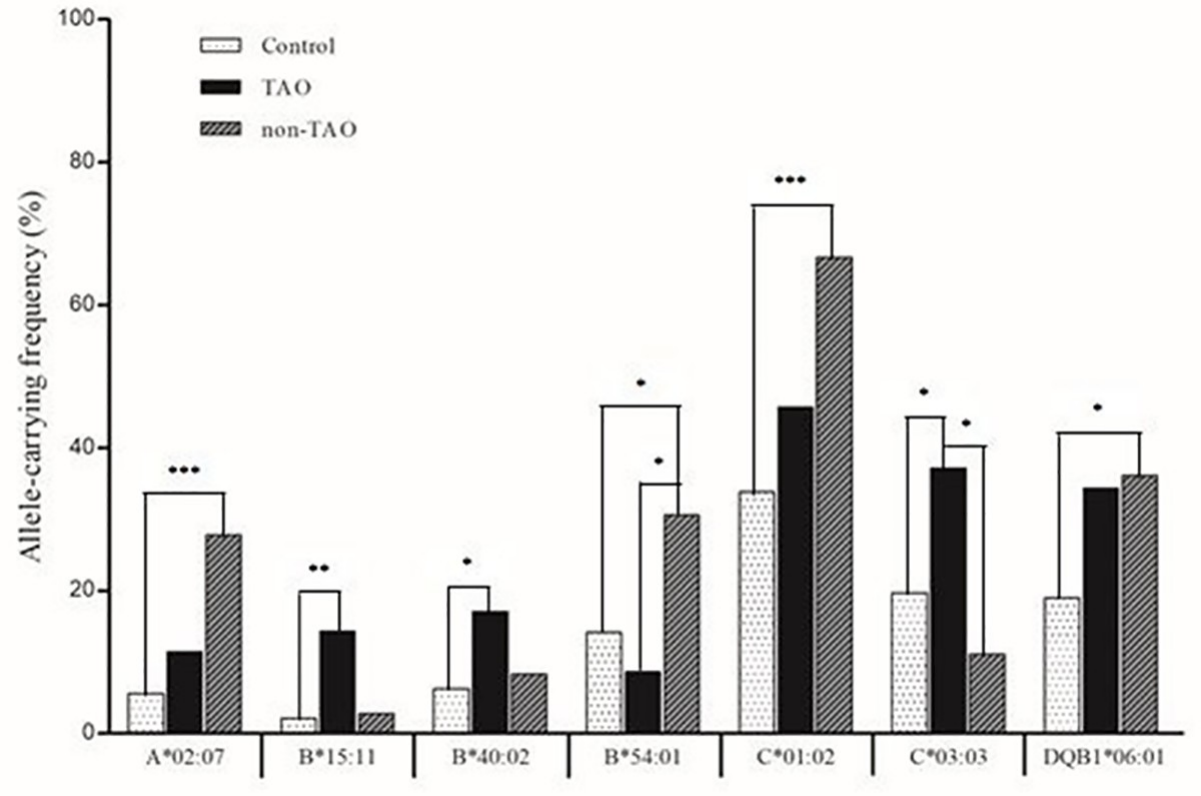
Distribution of HLA alleles showing differences between TAO and non-TAO (*P* < 0.05). HLA-C*03:03 (TAO vs. non-TAO; 37.1% vs. 11.1%, *P* = 0.02) showed higher frequency in TAO than non-TAO, whereas HLA-B*54:01 (TAO vs. non-TAO; 8.6% vs. 30.6%, *P* = 0.04) showed higher frequency in non-TAO than TAO. * *P* < 0.05; ** *P* < 0.01; *** *P* < 0.001.

In the analysis of associations of HLA class I (A, B, and C) alleles with TAO, significant positive associations were found for HLA-B*15:11 (OR = 7.19), -B*40:02 (OR = 3.1), and -C*03:03 (OR = 2.41) (*P* < 0.05) (Fig 1). Furthermore, HLA-A*02:07 (OR = 6.27), -B*54:01 (OR = 2.7), and -C*01:02 (OR = 3.82) showed positive associations with non-TAO (*P* < 0.05). Additionally, of the HLA class II (DRB1, DQB1, and DPB1) alleles, only HLA-DQB1*06:01 allele was positively associated with non-TAO. However, only the HLA class I alleles HLA-B*54:01 and -C*03:03 showed a significant difference according to the presence of TAO (*P* < 0.05).

### Associations of amino acid variants of HLA class II genes with AITD

Based on genotyping, amino acid variants of HLA class I and class II genes were analyzed to identify associations with AITD (Table 3). Amino acid variants of HLA-B (Lys66, Arg69, Val76), -C (Tyr116), -DRB1 (Ser57 and Leu74), and -DPB1 (Leu35 and Glu55) were significantly associated with GD. Leu35 and Glu55 of HLA-DPB1, which had the same *P*-value and OR, had the strongest association (OR = 23.38, *P* = 0.0002). In a comparison between GD and HD patients, they also showed significant differences at the same level (*P* = 0.001). In addition to the above amino acid variants, Cys99 of HLA-A (OR = 4.19, *P* = 0.0008), and Asp57 of HLA-DQB1 (OR = 10.91, *P* = 0.0005) were positively associated with only AITD (data not presented in the tables).

**Table 3:** Amino acid variants of HLA-DRB1, -DQB1 and -DPB1 showing higher significant associations with GD or with HD compared to those of HLA genotype in Korean children (*P* < 0.001).

		Amino acid		Controls		AITD		GD		Controls vs GD		HD		Controls vs HD	
Allele*	Locus	No.	Variant	n = 142 (%)		n = 116 (%)		n = 71 (%)		OR (95% CI)	p value	n = 45 (%)		OR (95% CI)	p value
46:01	B	66	Lys	16	(11.3)	35	(30.2)	24	(33.8)	3.96 (1.94–8.09)	0.0002	11	(24.4)		
46:01	B	69	Arg	16	(11.3)	35	(30.2)	24	(33.8)	3.96 (1.94–8.09)	0.0002	11	(24.4)		
46:01	B	76	Val	16	(11.3)	35	(30.2)	24	(33.8)	3.96 (1.94–8.09)	0.0002	11	(24.4)		
01:02	C	116	Tyr	82	(57.7)	95	(81.9)	59	(83.1)	3.49 (1.74–7.02)	0.0005	36	(80.0)		
08:03	DRB1	57	Ser	36	(25.4)	57	(49.1)	37	(52.1)	3.17 (1.74–5.77)	0.0002	20	(44.4)		
08:03, 14:03	DRB1	74	Leu	30	(21.1)	51	(44.0)	34	(47.9)	3.39 (1.83–6.28)	< .0001	17	(37.8)		
02:02, 05:01	DPB1	35	Leu	95	(66.9)	102	(87.9)	70	(98.6)	23.38 (4.40–124.32)	0.0002	32	(71.1)		
02:02, 05:01	DPB1	55	Glu	95	(66.9)	102	(87.9)	70	(98.6)	23.38 (4.40–124.32)	0.0002	32	(71.1)		

AITD, autoimmune diseases; GD, Graves' disease; HD, Hashimoto's disease; OR, Odds ratio; CI, Confidence intervals.

* The respective alleles in each implicated loci

### HLA class I and class II haplotypes associated with AITD

In the analysis of associations of HLA class I and class II haplotypes with GD, the haplotypes HLA-A*02:07-B*46:01-C*01:02, -A*02:06-B*46:01-C*01:02, -DRB1*04:05-DQB1*04:01-DPB1*05:01, -DRB1*08:03-DQB1*06:01-DPB1*02:02, and -DRB1*14:03-DQB1*03:01-DPB1*05:01 showed positive associations (*P* < 0.05) (Table 4). On the contrary, the haplotypes HLA-A*02:07-B*46:01-C*01:02, -DRB1*08:03-DQB1*06:01-DPB1*02:02, and -DRB1*09:01-DQB1*03:03-DPB1*02:01 were positively associated with HD (*P* < 0.05). For the six loci (HLA-A, -B, -C, -DRB1, -DQB1, and -DPB1), only HLA-A*02:07-*B46:01-C*01:02-DRB1*08:03-DQB1*06:01-DPB1*05:01 was positively associated with GD. In addition to the above haplotypes, HLA-A*30:01-B*13:02-C*06:02, A*33:03-B*44:03-C*14:03, and -DRB1*01:01-DQB1*05:01-DPB1*05:01 were negatively associated with only AITD. In the sex-adjusted analysis (*P* < 0.05), HLA-A*24:02-B*15:27-C*04:01, -A*26:01-B*54:01-C*01:02, -DRB1*04:06-DQB1*03:02-DPB1*03:01, -DRB1*14:54-DQB1*05:03-DPB1*02:02, -A*24:02-*B15:27-C*04:01-DRB1*11:01-DQB1*03:01-DPB1*03:01, -A*26:01-*B54:01-C*01:02-DRB1*14:54-DQB1*03:01-DPB1*04:01, and -A*33:03-*B58:01-C*03:02-DRB1*04:01-DQB1*05:03-DPB1*02:02 showed significant associations (*P* < 0.05) in HD patients (data of the sex-adjusted analysis not presented due to the absence of significant differences). Furthermore, haplotypes other than HLA-A*02:07-B*46:01-C*01:02 and DRB1*08:03-DQB1*06:01-DPB1*02:02 were differentially associated with GD and HD, respectively.

**Table 4:** Allele-carrying frequencies of HLA-A, -B, -C, -DRB1, -DQB1 and -DPB1 haplotypes significantly associated with GD or with HD in Korean children with AITD (*P* < 0.05).

	Controls		AITD		GD		Controls vs GD		HD		Controls vs HD	
Haplotypes	n = 142 (%)		n = 116 (%)		n = 71 (%)		OR (95% CI)	p value	n = 45 (%)		OR (95% CI)	p value
Class I (A, B ,C)												
02:06–46:01–01:02	2	(1.4)	9	(7.8)	8	(11.3)	7.52 (1.66–33.98)	0.009	1	(2.2)		
02:07–46:01–01:02	7	(4.9)	20	(17.2)	12	(16.9)	3.80 (1.43–10.09)	0.008	8	(17.8)	4.09 (1.40–12.02)	0.01
Class II (DRB1, DQB1, DPB1)												
04:05–04:01–05:01	11	(7.7)	20	(17.2)	14	(19.7)	2.88 (1.24–6.74)	0.01	6	(13.3)		
08:03–06:01–02:02	1	(0.7)	14	(12.1)	10	(14.1)	16.11 (2.63–98.62)	0.003	4	(8.9)	10.22 (1.31–79.69)	0.03
09:01–03:03–02:01	8	(5.6)	11	(9.5)	4	(5.6)			7	(15.6)	3.08 (1.05–9.04)	0.04
14:03–03:01–05:01	2	(1.4)	9	(7.8)	8	(11.3)	7.52 (1.66–33.98)	0.009	1	(2.2)		
Class I and II (A, B, C, DRB1, DQB1, DPB)												
02:07–46:01–01:02–08:03–06:01–05:01	5	(3.5)	10	(8.6)	9	(12.7)	3.80 (1.23–11.73)	0.02	1	(2.2)		

AITD, autoimmune diseases; GD, Graves’ disease; HD, Hashimoto’s disease; OR, odds ratio; CI, Confidence intervals.

## Discussion

Autoimmune responses to thyroid auto-antigenic peptides can occur because specific HLA pocket variants may allow auto-antigenic peptides to match the antigen-binding pocket of the HLA molecule and to be recognized by T cell receptors [23]. In our analysis of HLA amino acid variants associated with GD and HD (Table 3), certain amino acid variants in the HLA class I and II genes showed more significant associations than those of the HLA alleles. In Chinese patients with GD, Lys66, Arg69, and Val76 of HLA-B were identified, which were present in HLA-B*46:01 in our data. Tyr116 of HLA-C, which was present in HLA-C*01:02 in our data, was also proven to be associated with GD in Chinese [24]. Ser57 of HLA-DRB1, which was associated with GD in our data, has been reported to be associated with Primary Sjogren's syndrome in a Chinese study [25]. Furthermore, Leu74 of HLA-DRB1 was identified in Japanese patients with GD and rheumatoid arthritis [14, 26]. Interestingly, Asp57 of HLA-DQB1 was found to be strongly associated with AITD in our analysis of amino acid variants (data not presented in the tables), even though the DQB1 alleles did not show a significant positive association. Furthermore, Asp57 of HLA-DQB1 was also proven to be associated with GD in South Indian [27]. Finally, the strongest association with GD was observed in Leu35 and Glu55 of HLA-DPB1. Leu35 and Glu55 are both present in HLA-DPB1*02:02 and -DPB1*05:01, which have been found to be associated with GD (S1 Fig). The charge of the Glu-Ala residues at codon 55 and 56 differs from that of the other HLA-DPB1 alleles, which have Ala-Ala and Asp-Glu residues and are not associated with AITD [28]. Therefore, Glu55-Ala56 may be a crucial epitope on the DPβ chain for GD susceptibility that enables the binding of GD-associated peptides. In addition, Lys69 is present in HLA-DPBI*05:01, but Glu69 is present in DPB1*02:02, suggesting that Lys69 may also play an important role in susceptibility. Therefore, the combination of Glu55-Ala56 and Lys69 in the HLA-DPB1 molecule may be a major determinant of susceptibility to GD because those amino acids enable recognition of the groove of HLA class II molecules and are involved in binding of antigenic peptides [29]. An association of Leu35 and Glu55 with GD was identified in Japanese patients by applying a population specific HLA imputation panel [14]. In our comparison between GD and HD patients, we found that only Leu35 and Glu55 showed significant differences. These data, particularly regarding HLA class II genes, suggest that antigen-presentation ability or protein stability may make functional contributions to disease susceptibility.

In this study, the HLA-A*02:07 and -DPB1*02:02 were strongly associated with both GD and HD (Table 2). Furthermore, the HLA-A*02:07-*B46:01-C*01:02 and HLA-DRB1*08:03-DQB1*06:01-DPB1*02:02 haplotypes were also associated with both GD and HD (Table 4). A strong association between HLA-DPB1*02:02 and AITD was found in this study for the first time. In a previous Japanese study, GD patients without thyrotropin-binding inhibitory immunoglobulin showed an association with HLA-DPB1*0202 [30]. HLA-A2 has been found in various populations with an allele frequency of 10–40%. The distribution of HLA-A2 subtypes is distinctly different among different populations [31]. HLA-A*02:07 was found to be associated with GD in Taiwanese patients [32], and with HD in Japanese patients [6]. The major subtype HLA-A*02:01 has a different amino acid variant (Tyr99) compared to that of HLA-A*02:07 [33]. The HLA-DPB1*05:01 showed significantly different distributions between GD and HD (Table 2). This allele had the most significant difference, and has been reported to be associated with GD in Japanese [6].

HLA-B*46:01, -C*01:02, -DRB1*08:03, -DRB1*14:03, and -DPB1*05:01 showed significant positive associations with GD only (Table 2). Among the HLA class I alleles, HLA-B*46:01 is well known to be associated with GD. The association of HLA-B*46:01 with GD was consistent with Japanese and Chinese studies [34]. Similar to our previous data, HLA-B showed a significantly positive association with GD in patients with early-onset AITD (Table 2). HLA-C*01:02 was associated with GD in our data (Table 2). The same result was previously found in a Korean study [10], whereas HLA-C*0303 in Japanese [6] and -C*07 in Caucasians [35] were found to be associated with GD. An association of HLA-DRB1*08:03 and -DRB1*14:03 with GD was previously reported in studies of Korean [8, 9] and Japanese [6] patients with GD, whereas HLA-DRB1*0405, which was associated with early-onset GD in Japanese patients, was not found in our study. No association was found for HLA-DQB1 in our study, although HLA-DQB1*06:04 in Japanese [6] and -DQB1*0301 in Caucasians [36] were associated with GD. In cases of early-onset GD, HLA-DQB1*0303 in Chinese patients and -DQB1*0401 in Japanese patients were associated with GD [37, 38].

Because of the importance of TAO, we further identified HLA alleles showing differences between TAO (thyroid associated ophthalmopathy) and non-TAO (Fig 1). HLA-C*03:03 showed a higher frequency in patients with TAO than in those with non-TAO, whereas HLA-B*54:01 showed a higher frequency in patients with non-TAO than in those with TAO. Because HLA-B*54:01 and -C*03:03 were not associated with GD, they may play a role in the pathogenesis of Graves’ ophthalmopathy. The frequency of HLA-B54 was significantly higher in patients without ophthalmopathy than in those with severe ophthalmopathy in a Japanese study [39].

This study is meaningful both because it is one of the few studies of the six loci of HLA class I and II genes in AITD patients and because we tested and analyzed amino acid variants to gain a deeper understanding of the associations of HLA with AITD. The associations of HLA alleles and amino acid variants with the disease groups of AITD suggest that HLA molecules, especially HLA-DPB1*02:02 and -DPB1*05:01, may play a pivotal role in the molecular pathogenesis of early-onset AITD. To develop a deeper understanding of the direct molecular pathogenesis, it is necessary to investigate the role of HLA amino acid variants in autoantigen presentation. Furthermore, the fact that significant results were obtained even though a small number of subjects were studied suggests that the role of genetics may be stronger in early-onset AITD than in late-onset AITD. To better understand the genetic and environmental impacts of AITD onset, the difference between early-onset and late-onset AITD must be elucidated through direct comparisons in the future.

## Supporting information

S1 Table Allele-carrying frequencies of HLA-A, -B, -C, -DRB1, -DQB1 and -DPB1 alleles associated with GD or HD in Korean children with AITD (*P* < 0.05).
(DOCX)

S1 Fig Three-dimensional ribbon models of the HLA-DPB1 proteins associated with Graves’ disease risk. The protein structures of HLA-DPB1 is based on Protein Data Bank (PDB) (https://www.rcsb.org/) entries 3LQZ, which were prepared using UCSF (University of California, San Francisco) Chimera version 1.7. Residues at the Graves’ disease risk-associated amino acid positions are highlighted as colored spheres.
(TIF)
