# Supplementary material for: HLA alleles, especially amino-acid signatures of HLA-DPB1, might contribute to the molecular pathogenesis of early-onset autoimmune thyroid disease
Source: PLoS One. 2019 May 15;14(5):e0216941. doi: 10.1371/journal.pone.0216941 (PMC6519818; doi:10.1371/journal.pone.0216941)
Supplement: S1 Table — Allele-carrying frequencies of HLA-A, -B, -C, -DRB1, -DQB1 and -DPB1 alleles associated with GD or HD in Korean children with AITD (P < 0.05). (DOCX) [file pone.0216941.s001.docx]

**S1 Table. Allele-carrying frequencies of HLA-A, -B, -C, -DRB1, -DQB1 and -DPB1 alleles associated with GD or HD in Korean children with AITD (*P* < 0.05)**

|  |  |  | Controls | |  | AITD | | Controls vs AITD | | |  | GD | | Controls vs GD | | |  | HD | | Controls vs HD | | |  | GD vs HD | |
| --- | --- | --- | --- | --- | --- | --- | --- | --- | --- | --- | --- | --- | --- | --- | --- | --- | --- | --- | --- | --- | --- | --- | --- | --- | --- |
| Locus | Alleles |  | n = 142 (%) | |  | n = 116 (%) | | OR (95% CI) | p value | Pc |  | n = 71 (%) | | OR (95% CI) | p value | Pc |  | n = 45 (%) | | OR (95% CI) | p value | Pc |  | p value | Pc |
| A | 01:01 |  | 4 | (2.8) |  | 1 | (0.9) | 0.40 (0.05-3.07) | 0.38 |  |  | 1 | (1.4) | 0.66 (0.09-5.06) | 0.69 |  |  | 0 | (0.0) | 0.34 (0.01-9.03) | 0.52 |  |  | 0.77 |  |
| A | 02:01 |  | 53 | (37.3) |  | 35 | (30.2) | 0.73 (0.43-1.23) | 0.23 |  |  | 24 | (33.8) | 0.86 (0.48-1.57) | 0.63 |  |  | 11 | (24.4) | 0.56 (0.26-1.19) | 0.13 |  |  | 0.31 |  |
| A | 02:03 |  | 5 | (3.5) |  | 1 | (0.9) | 0.33 (0.05-2.32) | 0.26 |  |  | 1 | (1.4) | 0.53 (0.07-3.82) | 0.53 |  |  | 0 | (0.0) | 0.27 (0.01-6.67) | 0.43 |  |  | 0.77 |  |
| A | 02:06 |  | 24 | (16.9) |  | 24 | (20.7) | 1.28 (0.68-2.40) | 0.44 |  |  | 16 | (22.5) | 1.44 (0.71-2.92) | 0.31 |  |  | 8 | (17.8) | 1.10 (0.46-2.63) | 0.84 |  |  | 0.57 |  |
| A | 02:07 |  | 8 | (5.6) |  | 24 | (20.7) | 4.19 (1.82-9.67) | 0.0008 | 0.01 |  | 14 | (19.7) | 3.99 (1.59-10.01) | 0.003 |  |  | 10 | (22.2) | 4.68 (1.72-12.73) | 0.003 | 0.045 |  | 0.73 |  |
| A | 03:01 |  | 2 | (1.4) |  | 7 | (6.0) | 3.85 (0.83-17.78) | 0.08 |  |  | 3 | (4.2) | 2.87 (0.47-17.41) | 0.25 |  |  | 4 | (8.9) | 6.09 (1.11-33.54) | 0.04 |  |  | 0.34 |  |
| A | 11:01 |  | 33 | (23.2) |  | 25 | (21.6) | 0.91 (0.51-1.64) | 0.76 |  |  | 13 | (18.3) | 0.75 (0.37-1.54) | 0.44 |  |  | 12 | (26.7) | 1.22 (0.57-2.62) | 0.61 |  |  | 0.29 |  |
| A | 11:02 |  | 1 | (0.7) |  | 1 | (0.9) | 1.22 (0.08-19.79) | 0.89 |  |  | 1 | (1.4) | 2.01 (0.12-32.54) | 0.62 |  |  | 0 | (0.0) | 1.06 (0.01-96.78) | 0.98 |  |  | 0.77 |  |
| A | 24:02 |  | 46 | (32.4) |  | 50 | (43.1) | 1.58 (0.95-2.62) | 0.08 |  |  | 31 | (43.7) | 1.61 (0.90-2.90) | 0.11 |  |  | 19 | (42.2) | 1.53 (0.77-3.03) | 0.23 |  |  | 0.89 |  |
| A | 24:08 |  | 0 | (0.0) |  | 1 | (0.9) | 3.66 (0.04-336.73) | 0.57 |  |  | 1 | (1.4) | 6.29 (0.07-612.43) | 0.43 |  |  | 0 | (0.0) | - | - |  |  | 0.77 |  |
| A | 26:01 |  | 9 | (6.3) |  | 11 | (9.5) | 1.53 (0.61-3.83) | 0.36 |  |  | 6 | (8.5) | 1.40 (0.48-4.08) | 0.54 |  |  | 5 | (11.1) | 1.91 (0.61-5.98) | 0.27 |  |  | 0.62 |  |
| A | 26:02 |  | 15 | (10.6) |  | 4 | (3.4) | 0.33 (0.11-1.00) | 0.05 |  |  | 3 | (4.2) | 0.42 (0.12-1.43) | 0.17 |  |  | 1 | (2.2) | 0.28 (0.05-1.61) | 0.15 |  |  | 0.71 |  |
| A | 26:03 |  | 1 | (0.7) |  | 1 | (0.9) | 1.22 (0.08-19.79) | 0.89 |  |  | 1 | (1.4) | 2.01 (0.12-32.54) | 0.62 |  |  | 0 | (0.0) | 1.06 (0.01-96.78) | 0.98 |  |  | 0.77 |  |
| A | 30:01 |  | 12 | (8.5) |  | 2 | (1.7) | 0.23 (0.06-0.95) | 0.04 |  |  | 2 | (2.8) | 0.38 (0.09-1.58) | 0.18 |  |  | 0 | (0.0) | 0.12 (0.01-2.22) | 0.15 |  |  | 0.53 |  |
| A | 30:04 |  | 9 | (6.3) |  | 2 | (1.7) | 0.31 (0.07-1.34) | 0.12 |  |  | 2 | (2.8) | 0.51 (0.12-2.23) | 0.37 |  |  | 0 | (0.0) | 0.16 (0.01-3.15) | 0.22 |  |  | 0.53 |  |
| A | 31:01 |  | 11 | (7.7) |  | 10 | (8.6) | 1.13 (0.46-2.76) | 0.79 |  |  | 7 | (9.9) | 1.33 (0.49-3.58) | 0.57 |  |  | 3 | (6.7) | 0.94 (0.26-3.41) | 0.93 |  |  | 0.63 |  |
| A | 32:01 |  | 1 | (0.7) |  | 2 | (1.7) | 2.06 (0.20-21.58) | 0.55 |  |  | 1 | (1.4) | 2.01 (0.12-32.54) | 0.62 |  |  | 1 | (2.2) | 3.18 (0.20-51.92) | 0.42 |  |  | 0.75 |  |
| A | 33:03 |  | 38 | (26.8) |  | 18 | (15.5) | 0.51 (0.27-0.95) | 0.03 |  |  | 8 | (11.3) | 0.36 (0.16-0.82) | 0.01 |  |  | 10 | (22.2) | 0.80 (0.37-1.77) | 0.58 |  |  | 0.13 |  |
| B | 07:02 |  | 13 | (9.2) |  | 3 | (2.6) | 0.30 (0.09-1.02) | 0.05 |  |  | 2 | (2.8) | 0.35 (0.08-1.43) | 0.14 |  |  | 1 | (2.2) | 0.32 (0.06-1.92) | 0.21 |  |  | 0.96 |  |
| B | 08:01 |  | 2 | (1.4) |  | 1 | (0.9) | 0.73 (0.07-7.66) | 0.79 |  |  | 1 | (1.4) | 1.20 (0.11-12.61) | 0.88 |  |  | 0 | (0.0) | 0.62 (0.02-25.84) | 0.80 |  |  | 0.77 |  |
| B | 13:01 |  | 4 | (2.8) |  | 3 | (2.6) | 0.95 (0.21-4.31) | 0.95 |  |  | 0 | (0.0) | 0.22 (0.01-5.71) | 0.36 |  |  | 3 | (6.7) | 2.54 (0.55-11.74) | 0.23 |  |  | 0.16 |  |
| B | 13:02 |  | 13 | (9.2) |  | 2 | (1.7) | 0.21 (0.05-0.86) | 0.03 |  |  | 1 | (1.4) | 0.20 (0.04-1.20) | 0.08 |  |  | 1 | (2.2) | 0.32 (0.06-1.92) | 0.21 |  |  | 0.75 |  |
| B | 14:01 |  | 7 | (4.9) |  | 1 | (0.9) | 0.24 (0.04-1.53) | 0.13 |  |  | 1 | (1.4) | 0.39 (0.06-2.52) | 0.32 |  |  | 0 | (0.0) | 0.20 (0.01-4.31) | 0.30 |  |  | 0.77 |  |
| B | 15:01 |  | 22 | (15.5) |  | 14 | (12.1) | 0.76 (0.37-1.56) | 0.45 |  |  | 7 | (9.9) | 0.62 (0.26-1.52) | 0.30 |  |  | 7 | (15.6) | 1.04 (0.42-2.61) | 0.93 |  |  | 0.37 |  |
| B | 15:02 |  | 0 | (0.0) |  | 4 | (3.4) | 11.42 (0.43-302.82) | 0.15 |  |  | 1 | (1.4) | 6.29 (0.07-612.43) | 0.43 |  |  | 3 | (6.7) | 23.45 (0.75-729.54) | 0.07 |  |  | 0.22 |  |
| B | 15:07 |  | 3 | (2.1) |  | 1 | (0.9) | 0.52 (0.06-4.46) | 0.55 |  |  | 0 | (0.0) | 0.28 (0.01-8.64) | 0.47 |  |  | 1 | (2.2) | 1.34 (0.15-11.71) | 0.79 |  |  | 0.50 |  |
| B | 15:11 |  | 3 | (2.1) |  | 9 | (7.8) | 3.52 (0.96-12.93) | 0.06 |  |  | 6 | (8.5) | 3.95 (0.97-16.05) | 0.05 |  |  | 3 | (6.7) | 3.28 (0.64-16.88) | 0.15 |  |  | 0.80 |  |
| B | 15:18 |  | 5 | (3.5) |  | 3 | (2.6) | 0.77 (0.18-3.26) | 0.72 |  |  | 1 | (1.4) | 0.53 (0.07-3.82) | 0.53 |  |  | 2 | (4.4) | 1.44 (0.28-7.36) | 0.66 |  |  | 0.41 |  |
| B | 15:27 |  | 0 | (0.0) |  | 1 | (0.9) | 3.66 (0.04-336.73) | 0.57 |  |  | 0 | (0.0) | - | - |  |  | 1 | (2.2) | 9.71 (0.10-920.58) | 0.33 |  |  | 0.50 |  |
| B | 27:04 |  | 1 | (0.7) |  | 0 | (0.0) | 0.39 (0.004-37.87) | 0.69 |  |  | 0 | (0.0) | 0.64 (0.01-61.83) | 0.85 |  |  | 0 | (0.0) | 1.06 (0.01-96.78) | 0.98 |  |  | - |  |
| B | 27:05 |  | 10 | (7.0) |  | 3 | (2.6) | 0.39 (0.11-1.40) | 0.15 |  |  | 2 | (2.8) | 0.45 (0.11-1.96) | 0.29 |  |  | 1 | (2.2) | 0.43 (0.07-2.62) | 0.36 |  |  | 0.96 |  |
| B | 27:20 |  | 0 | (0.0) |  | 1 | (0.9) | 3.66 (0.04-336.73) | 0.57 |  |  | 1 | (1.4) | 6.29 (0.07-612.43) | 0.43 |  |  | 0 | (0.0) | - | - |  |  | 0.77 |  |
| B | 35:01 |  | 15 | (10.6) |  | 17 | (14.7) | 1.45 (0.69-3.04) | 0.33 |  |  | 11 | (15.5) | 1.56 (0.68-3.61) | 0.29 |  |  | 6 | (13.3) | 1.35 (0.50-3.69) | 0.55 |  |  | 0.79 |  |
| B | 35:03 |  | 0 | (0.0) |  | 1 | (0.9) | 3.66 (0.04-336.73) | 0.57 |  |  | 0 | (0.0) | - | - |  |  | 1 | (2.2) | 9.71 (0.10-920.58) | 0.33 |  |  | 0.50 |  |
| B | 37:01 |  | 5 | (3.5) |  | 1 | (0.9) | 0.33 (0.05-2.32) | 0.26 |  |  | 1 | (1.4) | 0.53 (0.07-3.82) | 0.53 |  |  | 0 | (0.0) | 0.27 (0.01-6.67) | 0.43 |  |  | 0.77 |  |
| B | 38:02 |  | 6 | (4.2) |  | 1 | (0.9) | 0.27 (0.04-1.85) | 0.18 |  |  | 1 | (1.4) | 0.45 (0.07-3.05) | 0.41 |  |  | 0 | (0.0) | 0.23 (0.01-5.25) | 0.36 |  |  | 0.77 |  |
| B | 39:01 |  | 2 | (1.4) |  | 1 | (0.9) | 0.73 (0.07-7.66) | 0.79 |  |  | 0 | (0.0) | 0.39 (0.01-16.38) | 0.62 |  |  | 1 | (2.2) | 1.90 (0.18-20.10) | 0.60 |  |  | 0.50 |  |
| B | 40:01 |  | 10 | (7.0) |  | 6 | (5.2) | 0.74 (0.26-2.10) | 0.57 |  |  | 5 | (7.0) | 1.04 (0.35-3.15) | 0.94 |  |  | 1 | (2.2) | 0.43 (0.07-2.62) | 0.36 |  |  | 0.38 |  |
| B | 40:02 |  | 9 | (6.3) |  | 10 | (8.6) | 1.39 (0.54-3.53) | 0.49 |  |  | 9 | (12.7) | 2.14 (0.81-5.65) | 0.13 |  |  | 1 | (2.2) | 0.47 (0.08-2.98) | 0.43 |  |  | 0.11 |  |
| B | 40:03 |  | 2 | (1.4) |  | 1 | (0.9) | 0.73 (0.07-7.66) | 0.79 |  |  | 1 | (1.4) | 1.20 (0.11-12.61) | 0.88 |  |  | 0 | (0.0) | 0.62 (0.02-25.84) | 0.80 |  |  | 0.77 |  |
| B | 40:06 |  | 10 | (7.0) |  | 9 | (7.8) | 1.12 (0.44-2.84) | 0.82 |  |  | 4 | (5.6) | 0.84 (0.26-2.74) | 0.77 |  |  | 5 | (11.1) | 1.71 (0.56-5.27) | 0.35 |  |  | 0.31 |  |
| B | 44:02 |  | 3 | (2.1) |  | 8 | (6.9) | 3.12 (0.83-11.75) | 0.09 |  |  | 4 | (5.6) | 2.66 (0.58-12.17) | 0.21 |  |  | 4 | (8.9) | 4.32 (0.93-20.03) | 0.06 |  |  | 0.51 |  |
| B | 44:03 |  | 19 | (13.4) |  | 2 | (1.7) | 0.14 (0.04-0.55) | 0.005 |  |  | 1 | (1.4) | 0.14 (0.02-0.76) | 0.02 |  |  | 1 | (2.2) | 0.21 (0.04-1.21) | 0.08 |  |  | 0.75 |  |
| B | 46:01 |  | 16 | (11.3) |  | 35 | (30.2) | 3.34 (1.74-6.41) | 0.0003 | 0.01 |  | 24 | (33.8) | 3.96 (1.94-8.09) | 0.0002 | 0.008 |  | 11 | (24.4) | 2.56 (1.09-6.01) | 0.03 |  |  | 0.31 |  |
| B | 48:01 |  | 15 | (10.6) |  | 7 | (6.0) | 0.56 (0.22-1.42) | 0.23 |  |  | 3 | (4.2) | 0.42 (0.12-1.43) | 0.17 |  |  | 4 | (8.9) | 0.89 (0.29-2.77) | 0.84 |  |  | 0.34 |  |
| B | 51:01 |  | 24 | (16.9) |  | 21 | (18.1) | 1.09 (0.57-2.08) | 0.80 |  |  | 16 | (22.5) | 1.44 (0.71-2.92) | 0.31 |  |  | 5 | (11.1) | 0.66 (0.24-1.80) | 0.41 |  |  | 0.15 |  |
| B | 51:02 |  | 3 | (2.1) |  | 6 | (5.2) | 2.34 (0.58-9.43) | 0.23 |  |  | 3 | (4.2) | 2.04 (0.40-10.36) | 0.39 |  |  | 3 | (6.7) | 3.28 (0.64-16.88) | 0.15 |  |  | 0.57 |  |
| B | 52:01 |  | 5 | (3.5) |  | 3 | (2.6) | 0.77 (0.18-3.26) | 0.72 |  |  | 3 | (4.2) | 1.28 (0.30-5.45) | 0.74 |  |  | 0 | (0.0) | 0.27 (0.01-6.67) | 0.43 |  |  | 0.38 |  |
| B | 54:01 |  | 20 | (14.1) |  | 21 | (18.1) | 1.35 (0.69-2.63) | 0.38 |  |  | 14 | (19.7) | 1.51 (0.71-3.19) | 0.28 |  |  | 7 | (15.6) | 1.16 (0.46-2.94) | 0.75 |  |  | 0.61 |  |
| B | 55:02 |  | 6 | (4.2) |  | 3 | (2.6) | 0.65 (0.16-2.61) | 0.54 |  |  | 0 | (0.0) | 0.15 (0.01-3.33) | 0.23 |  |  | 3 | (6.7) | 1.73 (0.42-7.10) | 0.45 |  |  | 0.16 |  |
| B | 56:01 |  | 1 | (0.7) |  | 0 | (0.0) | 0.39 (0.004-37.87) | 0.69 |  |  | 0 | (0.0) | 0.64 (0.01-61.83) | 0.85 |  |  | 0 | (0.0) | 1.06 (0.01-96.78) | 0.98 |  |  | - |  |
| B | 56:05 |  | 0 | (0.0) |  | 1 | (0.9) | 3.66 (0.04-336.73) | 0.57 |  |  | 1 | (1.4) | 6.29 (0.07-612.43) | 0.43 |  |  | 0 | (0.0) | - | - |  |  | 0.77 |  |
| B | 57:01 |  | 1 | (0.7) |  | 0 | (0.0) | 0.39 (0.004-37.87) | 0.69 |  |  | 0 | (0.0) | 0.64 (0.01-61.83) | 0.85 |  |  | 0 | (0.0) | 1.06 (0.01-96.78) | 0.98 |  |  | - |  |
| B | 58:01 |  | 16 | (11.3) |  | 14 | (12.1) | 1.08 (0.51-2.33) | 0.84 |  |  | 7 | (9.9) | 0.89 (0.35-2.26) | 0.81 |  |  | 7 | (15.6) | 1.49 (0.58-3.87) | 0.41 |  |  | 0.37 |  |
| B | 59:01 |  | 3 | (2.1) |  | 2 | (1.7) | 0.87 (0.15-5.24) | 0.88 |  |  | 1 | (1.4) | 0.85 (0.10-7.34) | 0.88 |  |  | 1 | (2.2) | 1.34 (0.15-11.71) | 0.79 |  |  | 0.75 |  |
| B | 67:01 |  | 4 | (2.8) |  | 3 | (2.6) | 0.95 (0.21-4.31) | 0.95 |  |  | 2 | (2.8) | 1.11 (0.20-6.03) | 0.91 |  |  | 1 | (2.2) | 1.04 (0.13-8.08) | 0.97 |  |  | 0.96 |  |
| C | 01:02 |  | 48 | (33.8) |  | 63 | (54.3) | 2.31 (1.40-3.83) | 0.001 | 0.02 |  | 40 | (56.3) | 2.51 (1.40-4.49) | 0.002 | 0.04 |  | 23 | (51.1) | 2.04 (1.03-4.01) | 0.04 |  |  | 0.59 |  |
| C | 01:03 |  | 3 | (2.1) |  | 1 | (0.9) | 0.52 (0.06-4.46) | 0.55 |  |  | 0 | (0.0) | 0.28 (0.01-8.64) | 0.47 |  |  | 1 | (2.2) | 1.34 (0.15-11.71) | 0.79 |  |  | 0.50 |  |
| C | 02:02 |  | 1 | (0.7) |  | 1 | (0.9) | 1.22 (0.08-19.79) | 0.89 |  |  | 0 | (0.0) | 0.64 (0.01-61.83) | 0.85 |  |  | 1 | (2.2) | 3.18 (0.20-51.92) | 0.42 |  |  | 0.50 |  |
| C | 03:02 |  | 16 | (11.3) |  | 15 | (12.9) | 1.17 (0.55-2.48) | 0.68 |  |  | 7 | (9.9) | 0.89 (0.35-2.26) | 0.81 |  |  | 8 | (17.8) | 1.74 (0.69-4.36) | 0.24 |  |  | 0.23 |  |
| C | 03:03 |  | 28 | (19.7) |  | 26 | (22.4) | 1.18 (0.65-2.15) | 0.60 |  |  | 17 | (23.9) | 1.29 (0.65-2.55) | 0.47 |  |  | 9 | (20.0) | 1.05 (0.46-2.41) | 0.92 |  |  | 0.65 |  |
| C | 03:04 |  | 21 | (14.8) |  | 22 | (19.0) | 1.35 (0.70-2.59) | 0.38 |  |  | 15 | (21.1) | 1.55 (0.74-3.23) | 0.24 |  |  | 7 | (15.6) | 1.10 (0.44-2.76) | 0.84 |  |  | 0.49 |  |
| C | 04:01 |  | 14 | (9.9) |  | 12 | (10.3) | 1.06 (0.47-2.39) | 0.89 |  |  | 6 | (8.5) | 0.88 (0.33-2.38) | 0.80 |  |  | 6 | (13.3) | 1.46 (0.53-4.02) | 0.47 |  |  | 0.41 |  |
| C | 05:01 |  | 3 | (2.1) |  | 8 | (6.9) | 3.12 (0.83-11.75) | 0.09 |  |  | 4 | (5.6) | 2.66 (0.58-12.17) | 0.21 |  |  | 4 | (8.9) | 4.32 (0.93-20.03) | 0.06 |  |  | 0.51 |  |
| C | 06:02 |  | 18 | (12.7) |  | 2 | (1.7) | 0.15 (0.04-0.58) | 0.01 |  |  | 1 | (1.4) | 0.14 (0.03-0.81) | 0.03 |  |  | 1 | (2.2) | 0.23 (0.04-1.29) | 0.10 |  |  | 0.75 |  |
| C | 07:01 |  | 0 | (0.0) |  | 2 | (1.7) | 6.22 (0.15-257.87) | 0.34 |  |  | 1 | (1.4) | 6.29 (0.07-612.43) | 0.43 |  |  | 1 | (2.2) | 9.71 (0.10-920.58) | 0.33 |  |  | 0.75 |  |
| C | 07:02 |  | 25 | (17.6) |  | 9 | (7.8) | 0.41 (0.18-0.91) | 0.03 |  |  | 5 | (7.0) | 0.38 (0.14-1.02) | 0.05 |  |  | 4 | (8.9) | 0.50 (0.17-1.47) | 0.21 |  |  | 0.70 |  |
| C | 07:04 |  | 5 | (3.5) |  | 2 | (1.7) | 0.55 (0.11-2.75) | 0.46 |  |  | 0 | (0.0) | 0.18 (0.01-4.22) | 0.28 |  |  | 2 | (4.4) | 1.44 (0.28-7.36) | 0.66 |  |  | 0.27 |  |
| C | 07:06 |  | 6 | (4.2) |  | 0 | (0.0) | 0.09 (0.004-2.03) | 0.13 |  |  | 0 | (0.0) | 0.15 (0.01-3.33) | 0.23 |  |  | 0 | (0.0) | 0.23 (0.01-5.25) | 0.36 |  |  | - |  |
| C | 08:01 |  | 22 | (15.5) |  | 16 | (13.8) | 0.88 (0.44-1.76) | 0.72 |  |  | 9 | (12.7) | 0.81 (0.36-1.87) | 0.63 |  |  | 7 | (15.6) | 1.04 (0.42-2.61) | 0.93 |  |  | 0.65 |  |
| C | 08:02 |  | 7 | (4.9) |  | 1 | (0.9) | 0.24 (0.04-1.53) | 0.13 |  |  | 1 | (1.4) | 0.39 (0.06-2.52) | 0.32 |  |  | 0 | (0.0) | 0.20 (0.01-4.31) | 0.30 |  |  | 0.77 |  |
| C | 08:03 |  | 3 | (2.1) |  | 1 | (0.9) | 0.52 (0.06-4.46) | 0.55 |  |  | 0 | (0.0) | 0.28 (0.01-8.64) | 0.47 |  |  | 1 | (2.2) | 1.34 (0.15-11.71) | 0.79 |  |  | 0.50 |  |
| C | 12:02 |  | 6 | (4.2) |  | 4 | (3.4) | 0.84 (0.23-3.04) | 0.79 |  |  | 4 | (5.6) | 1.40 (0.38-5.11) | 0.61 |  |  | 0 | (0.0) | 0.23 (0.01-5.25) | 0.36 |  |  | 0.28 |  |
| C | 12:03 |  | 2 | (1.4) |  | 1 | (0.9) | 0.73 (0.07-7.66) | 0.79 |  |  | 0 | (0.0) | 0.39 (0.01-16.38) | 0.62 |  |  | 1 | (2.2) | 1.90 (0.18-20.10) | 0.60 |  |  | 0.50 |  |
| C | 14:02 |  | 21 | (14.8) |  | 21 | (18.1) | 1.27 (0.66-2.47) | 0.48 |  |  | 18 | (25.4) | 1.95 (0.96-3.96) | 0.06 |  |  | 3 | (6.7) | 0.47 (0.14-1.55) | 0.21 |  |  | 0.02 |  |
| C | 14:03 |  | 15 | (10.6) |  | 0 | (0.0) | 0.04 (0.002-0.65) | 0.02 |  |  | 0 | (0.0) | 0.06 (0.00-1.07) | 0.06 |  |  | 0 | (0.0) | 0.09 (0.01-1.69) | 0.11 |  |  | - |  |
| C | 15:02 |  | 8 | (5.6) |  | 8 | (6.9) | 1.24 (0.45-3.41) | 0.68 |  |  | 4 | (5.6) | 1.05 (0.31-3.59) | 0.93 |  |  | 4 | (8.9) | 1.72 (0.50-5.93) | 0.39 |  |  | 0.51 |  |
| DRB1 | 01:01 |  | 20 | (14.1) |  | 4 | (3.4) | 0.24 (0.08-0.70) | 0.009 |  |  | 3 | (4.2) | 0.31 (0.09-1.01) | 0.05 |  |  | 1 | (2.2) | 0.20 (0.04-1.14) | 0.07 |  |  | 0.71 |  |
| DRB1 | 03:01 |  | 8 | (5.6) |  | 7 | (6.0) | 1.08 (0.38-3.08) | 0.88 |  |  | 4 | (5.6) | 1.05 (0.31-3.59) | 0.93 |  |  | 3 | (6.7) | 1.30 (0.34-5.00) | 0.70 |  |  | 0.79 |  |
| DRB1 | 04:01 |  | 2 | (1.4) |  | 5 | (4.3) | 2.77 (0.55-13.97) | 0.22 |  |  | 2 | (2.8) | 2.02 (0.28-14.65) | 0.49 |  |  | 3 | (6.7) | 4.63 (0.76-28.29) | 0.10 |  |  | 0.37 |  |
| DRB1 | 04:03 |  | 8 | (5.6) |  | 5 | (4.3) | 0.78 (0.25-2.44) | 0.67 |  |  | 2 | (2.8) | 0.57 (0.13-2.57) | 0.46 |  |  | 3 | (6.7) | 1.30 (0.34-5.00) | 0.70 |  |  | 0.37 |  |
| DRB1 | 04:04 |  | 6 | (4.2) |  | 1 | (0.9) | 0.27 (0.04-1.85) | 0.18 |  |  | 1 | (1.4) | 0.45 (0.07-3.05) | 0.41 |  |  | 0 | (0.0) | 0.23 (0.01-5.25) | 0.36 |  |  | 0.77 |  |
| DRB1 | 04:05 |  | 16 | (11.3) |  | 26 | (22.4) | 2.24 (1.14-4.42) | 0.02 |  |  | 16 | (22.5) | 2.28 (1.06-4.88) | 0.03 |  |  | 10 | (22.2) | 2.27 (0.95-5.43) | 0.07 |  |  | 0.99 |  |
| DRB1 | 04:06 |  | 11 | (7.7) |  | 10 | (8.6) | 1.13 (0.46-2.76) | 0.79 |  |  | 6 | (8.5) | 1.13 (0.40-3.19) | 0.81 |  |  | 4 | (8.9) | 1.24 (0.38-4.03) | 0.72 |  |  | 0.90 |  |
| DRB1 | 04:07 |  | 3 | (2.1) |  | 1 | (0.9) | 0.52 (0.06-4.46) | 0.55 |  |  | 0 | (0.0) | 0.28 (0.01-8.64) | 0.47 |  |  | 1 | (2.2) | 1.34 (0.15-11.71) | 0.79 |  |  | 0.50 |  |
| DRB1 | 04:08 |  | 0 | (0.0) |  | 1 | (0.9) | 3.66 (0.04-336.73) | 0.57 |  |  | 0 | (0.0) | - | - |  |  | 1 | (2.2) | 9.71 (0.10-920.58) | 0.33 |  |  | 0.50 |  |
| DRB1 | 04:10 |  | 3 | (2.1) |  | 3 | (2.6) | 1.23 (0.24-6.21) | 0.80 |  |  | 3 | (4.2) | 2.04 (0.40-10.36) | 0.39 |  |  | 0 | (0.0) | 0.44 (0.01-13.64) | 0.64 |  |  | 0.38 |  |
| DRB1 | 07:01 |  | 19 | (13.4) |  | 4 | (3.4) | 0.25 (0.09-0.75) | 0.01 |  |  | 2 | (2.8) | 0.23 (0.06-0.91) | 0.04 |  |  | 2 | (4.4) | 0.36 (0.09-1.46) | 0.15 |  |  | 0.65 |  |
| DRB1 | 08:02 |  | 10 | (7.0) |  | 8 | (6.9) | 0.99 (0.38-2.59) | 0.98 |  |  | 5 | (7.0) | 1.04 (0.35-3.15) | 0.94 |  |  | 3 | (6.7) | 1.04 (0.28-3.82) | 0.95 |  |  | 0.99 |  |
| DRB1 | 08:03 |  | 20 | (14.1) |  | 35 | (30.2) | 2.60 (1.41-4.82) | 0.002 |  |  | 22 | (31.0) | 2.72 (1.36-5.42) | 0.005 |  |  | 13 | (28.9) | 2.48 (1.12-5.51) | 0.03 |  |  | 0.83 |  |
| DRB1 | 09:01 |  | 23 | (16.2) |  | 23 | (19.8) | 1.28 (0.68-2.42) | 0.45 |  |  | 10 | (14.1) | 0.87 (0.39-1.93) | 0.73 |  |  | 13 | (28.9) | 2.11 (0.97-4.62) | 0.06 |  |  | 0.06 |  |
| DRB1 | 10:01 |  | 4 | (2.8) |  | 1 | (0.9) | 0.40 (0.05-3.07) | 0.38 |  |  | 1 | (1.4) | 0.66 (0.09-5.06) | 0.69 |  |  | 0 | (0.0) | 0.34 (0.01-9.03) | 0.52 |  |  | 0.77 |  |
| DRB1 | 11:01 |  | 12 | (8.5) |  | 9 | (7.8) | 0.92 (0.38-2.27) | 0.86 |  |  | 4 | (5.6) | 0.70 (0.22-2.20) | 0.54 |  |  | 5 | (11.1) | 1.42 (0.48-4.22) | 0.53 |  |  | 0.31 |  |
| DRB1 | 11:06 |  | 1 | (0.7) |  | 0 | (0.0) | 0.39 (0.00-37.87) | 0.69 |  |  | 0 | (0.0) | 0.64 (0.01-61.83) | 0.85 |  |  | 0 | (0.0) | 1.06 (0.01-96.78) | 0.98 |  |  | - |  |
| DRB1 | 12:01 |  | 9 | (6.3) |  | 12 | (10.3) | 1.68 (0.68-4.14) | 0.26 |  |  | 9 | (12.7) | 2.14 (0.81-5.65) | 0.13 |  |  | 3 | (6.7) | 1.16 (0.31-4.34) | 0.83 |  |  | 0.37 |  |
| DRB1 | 12:02 |  | 10 | (7.0) |  | 6 | (5.2) | 0.74 (0.26-2.10) | 0.57 |  |  | 2 | (2.8) | 0.45 (0.11-1.96) | 0.29 |  |  | 4 | (8.9) | 1.37 (0.41-4.52) | 0.61 |  |  | 0.21 |  |
| DRB1 | 13:01 |  | 2 | (1.4) |  | 5 | (4.3) | 2.77 (0.55-13.97) | 0.22 |  |  | 3 | (4.2) | 2.87 (0.47-17.41) | 0.25 |  |  | 2 | (4.4) | 3.23 (0.44-23.62) | 0.25 |  |  | 0.90 |  |
| DRB1 | 13:02 |  | 23 | (16.2) |  | 6 | (5.2) | 0.30 (0.12-0.75) | 0.01 |  |  | 3 | (4.2) | 0.26 (0.08-0.85) | 0.03 |  |  | 3 | (6.7) | 0.42 (0.13-1.39) | 0.15 |  |  | 0.57 |  |
| DRB1 | 14:03 |  | 2 | (1.4) |  | 9 | (7.8) | 4.97 (1.13-21.74) | 0.03 |  |  | 8 | (11.3) | 7.52 (1.66-33.98) | 0.009 |  |  | 1 | (2.2) | 1.90 (0.18-20.10) | 0.60 |  |  | 0.15 |  |
| DRB1 | 14:04 |  | 1 | (0.7) |  | 1 | (0.9) | 1.22 (0.08-19.79) | 0.89 |  |  | 1 | (1.4) | 2.01 (0.12-32.54) | 0.62 |  |  | 0 | (0.0) | 1.06 (0.01-96.78) | 0.98 |  |  | 0.77 |  |
| DRB1 | 14:05 |  | 15 | (10.6) |  | 7 | (6.0) | 0.56 (0.22-1.42) | 0.23 |  |  | 4 | (5.6) | 0.55 (0.18-1.68) | 0.29 |  |  | 3 | (6.7) | 0.68 (0.20-2.35) | 0.54 |  |  | 0.79 |  |
| DRB1 | 14:06 |  | 4 | (2.8) |  | 1 | (0.9) | 0.40 (0.05-3.07) | 0.38 |  |  | 1 | (1.4) | 0.66 (0.09-5.06) | 0.69 |  |  | 0 | (0.0) | 0.34 (0.01-9.03) | 0.52 |  |  | 0.77 |  |
| DRB1 | 14:54 |  | 6 | (4.2) |  | 9 | (7.8) | 1.86 (0.64-5.36) | 0.25 |  |  | 6 | (8.5) | 2.08 (0.65-6.71) | 0.22 |  |  | 3 | (6.7) | 1.73 (0.42-7.10) | 0.45 |  |  | 0.80 |  |
| DRB1 | 15:01 |  | 21 | (14.8) |  | 13 | (11.2) | 0.74 (0.35-1.54) | 0.42 |  |  | 13 | (18.3) | 1.30 (0.61-2.78) | 0.49 |  |  | 0 | (0.0) | 0.06 (0.00-1.12) | 0.06 |  |  | 0.04 |  |
| DRB1 | 15:02 |  | 13 | (9.2) |  | 3 | (2.6) | 0.30 (0.09-1.02) | 0.05 |  |  | 3 | (4.2) | 0.49 (0.14-1.71) | 0.26 |  |  | 0 | (0.0) | 0.11 (0.01-2.01) | 0.13 |  |  | 0.38 |  |
| DRB1 | 16:02 |  | 5 | (3.5) |  | 1 | (0.9) | 0.33 (0.05-2.32) | 0.26 |  |  | 0 | (0.0) | 0.18 (0.01-4.22) | 0.28 |  |  | 1 | (2.2) | 0.84 (0.12-6.10) | 0.87 |  |  | 0.50 |  |
| DQB1 | 02:01 |  | 7 | (4.9) |  | 9 | (7.8) | 1.60 (0.58-4.42) | 0.37 |  |  | 5 | (7.0) | 1.50 (0.46-4.88) | 0.51 |  |  | 4 | (8.9) | 1.96 (0.55-6.97) | 0.30 |  |  | 0.70 |  |
| DQB1 | 02:02 |  | 17 | (12.0) |  | 1 | (0.9) | 0.09 (0.02-0.53) | 0.007 |  |  | 1 | (1.4) | 0.15 (0.03-0.87) | 0.03 |  |  | 0 | (0.0) | 0.08 (0.00-1.45) | 0.09 |  |  | 0.77 |  |
| DQB1 | 03:01 |  | 35 | (24.6) |  | 39 | (33.6) | 1.54 (0.90-2.65) | 0.12 |  |  | 23 | (32.4) | 1.47 (0.78-2.74) | 0.23 |  |  | 16 | (35.6) | 1.69 (0.83-3.47) | 0.15 |  |  | 0.72 |  |
| DQB1 | 03:02 |  | 30 | (21.1) |  | 19 | (16.4) | 0.74 (0.39-1.39) | 0.35 |  |  | 10 | (14.1) | 0.63 (0.29-1.37) | 0.24 |  |  | 9 | (20.0) | 0.96 (0.42-2.20) | 0.92 |  |  | 0.40 |  |
| DQB1 | 03:03 |  | 25 | (17.6) |  | 26 | (22.4) | 1.35 (0.73-2.49) | 0.34 |  |  | 12 | (16.9) | 0.97 (0.46-2.06) | 0.93 |  |  | 14 | (31.1) | 2.12 (0.99-4.55) | 0.05 |  |  | 0.08 |  |
| DQB1 | 04:01 |  | 17 | (12.0) |  | 26 | (22.4) | 2.10 (1.08-4.09) | 0.03 |  |  | 16 | (22.5) | 2.13 (1.01-4.53) | 0.05 |  |  | 10 | (22.2) | 2.12 (0.89-5.03) | 0.09 |  |  | 0.99 |  |
| DQB1 | 04:02 |  | 14 | (9.9) |  | 11 | (9.5) | 0.97 (0.42-2.22) | 0.94 |  |  | 8 | (11.3) | 1.19 (0.47-2.97) | 0.72 |  |  | 3 | (6.7) | 0.73 (0.21-2.55) | 0.62 |  |  | 0.48 |  |
| DQB1 | 05:01 |  | 26 | (18.3) |  | 5 | (4.3) | 0.22 (0.08-0.57) | 0.002 | 0.03 |  | 4 | (5.6) | 0.29 (0.10-0.85) | 0.02 |  |  | 1 | (2.2) | 0.15 (0.03-0.82) | 0.03 |  |  | 0.52 |  |
| DQB1 | 05:02 |  | 7 | (4.9) |  | 8 | (6.9) | 1.42 (0.50-4.03) | 0.51 |  |  | 6 | (8.5) | 1.79 (0.58-5.55) | 0.31 |  |  | 2 | (4.4) | 1.04 (0.22-4.88) | 0.96 |  |  | 0.50 |  |
| DQB1 | 05:03 |  | 19 | (13.4) |  | 12 | (10.3) | 0.76 (0.35-1.63) | 0.48 |  |  | 6 | (8.5) | 0.63 (0.24-1.63) | 0.34 |  |  | 6 | (13.3) | 1.04 (0.39-2.76) | 0.93 |  |  | 0.41 |  |
| DQB1 | 06:01 |  | 27 | (19.0) |  | 37 | (31.9) | 1.98 (1.12-3.51) | 0.02 |  |  | 25 | (35.2) | 2.30 (1.21-4.38) | 0.01 |  |  | 12 | (26.7) | 1.57 (0.72-3.42) | 0.26 |  |  | 0.36 |  |
| DQB1 | 06:02 |  | 21 | (14.8) |  | 13 | (11.2) | 0.74 (0.35-1.54) | 0.42 |  |  | 13 | (18.3) | 1.30 (0.61-2.78) | 0.49 |  |  | 0 | (0.0) | 0.06 (0.00-1.12) | 0.06 |  |  | 0.04 |  |
| DQB1 | 06:03 |  | 2 | (1.4) |  | 5 | (4.3) | 2.77 (0.55-13.97) | 0.22 |  |  | 3 | (4.2) | 2.87 (0.47-17.41) | 0.25 |  |  | 2 | (4.4) | 3.23 (0.44-23.62) | 0.25 |  |  | 0.90 |  |
| DQB1 | 06:04 |  | 16 | (11.3) |  | 0 | (0.0) | 0.03 (0.002-0.61) | 0.02 |  |  | 0 | (0.0) | 0.05 (0.00-0.99) | 0.05 |  |  | 0 | (0.0) | 0.08 (0.01-1.56) | 0.10 |  |  | - |  |
| DQB1 | 06:09 |  | 8 | (5.6) |  | 6 | (5.2) | 0.93 (0.31-2.76) | 0.90 |  |  | 3 | (4.2) | 0.81 (0.21-3.06) | 0.75 |  |  | 3 | (6.7) | 1.30 (0.34-5.00) | 0.70 |  |  | 0.57 |  |
| DPB1 | 02:01 |  | 64 | (45.1) |  | 49 | (42.2) | 0.89 (0.54-1.46) | 0.65 |  |  | 26 | (36.6) | 0.71 (0.40-1.27) | 0.25 |  |  | 23 | (51.1) | 1.27 (0.65-2.48) | 0.48 |  |  | 0.13 |  |
| DPB1 | 02:02 |  | 8 | (5.6) |  | 27 | (23.3) | 4.86 (2.13-11.09) | 0.0002 | 0.003 |  | 14 | (19.7) | 3.99 (1.59-10.01) | 0.003 | 0.04 |  | 13 | (28.9) | 6.57 (2.52-17.14) | 0.0001 | 0.001 |  | 0.26 |  |
| DPB1 | 03:01 |  | 7 | (4.9) |  | 10 | (8.6) | 1.78 (0.66-4.83) | 0.26 |  |  | 5 | (7.0) | 1.50 (0.46-4.88) | 0.51 |  |  | 5 | (11.1) | 2.45 (0.74-8.13) | 0.14 |  |  | 0.45 |  |
| DPB1 | 04:01 |  | 22 | (15.5) |  | 11 | (9.5) | 0.58 (0.27-1.26) | 0.17 |  |  | 3 | (4.2) | 0.27 (0.08-0.90) | 0.03 |  |  | 8 | (17.8) | 1.21 (0.50-2.93) | 0.67 |  |  | 0.03 |  |
| DPB1 | 04:02 |  | 21 | (14.8) |  | 15 | (12.9) | 0.86 (0.42-1.76) | 0.69 |  |  | 9 | (12.7) | 0.86 (0.37-1.98) | 0.72 |  |  | 6 | (13.3) | 0.93 (0.36-2.44) | 0.88 |  |  | 0.89 |  |
| DPB1 | 05:01 |  | 88 | (62.0) |  | 86 | (74.1) | 1.75 (1.02-2.99) | 0.04 |  |  | 63 | (88.7) | 4.60 (2.07-10.21) | 0.0002 | 0.003 |  | 23 | (51.1) | 0.64 (0.33-1.26) | 0.20 |  |  | 0.00004 | 0.0005 |
| DPB1 | 09:01 |  | 9 | (6.3) |  | 3 | (2.6) | 0.43 (0.12-1.59) | 0.21 |  |  | 3 | (4.2) | 0.72 (0.19-2.66) | 0.62 |  |  | 0 | (0.0) | 0.16 (0.01-3.15) | 0.22 |  |  | 0.38 |  |
| DPB1 | 13:01 |  | 19 | (13.4) |  | 7 | (6.0) | 0.43 (0.18-1.06) | 0.07 |  |  | 6 | (8.5) | 0.63 (0.24-1.63) | 0.34 |  |  | 1 | (2.2) | 0.21 (0.04-1.21) | 0.08 |  |  | 0.27 |  |
| DPB1 | 14:01 |  | 5 | (3.5) |  | 3 | (2.6) | 0.77 (0.18-3.26) | 0.72 |  |  | 0 | (0.0) | 0.18 (0.01-4.22) | 0.28 |  |  | 3 | (6.7) | 2.06 (0.48-8.89) | 0.33 |  |  | 0.16 |  |
| DPB1 | 17:01 |  | 9 | (6.3) |  | 0 | (0.0) | 0.06 (0.003-1.22) | 0.07 |  |  | 0 | (0.0) | 0.10 (0.01-2.00) | 0.13 |  |  | 0 | (0.0) | 0.16 (0.01-3.15) | 0.22 |  |  | - |  |
| DPB1 | 21:01 |  | 0 | (0.0) |  | 1 | (0.9) | 3.66 (0.04-336.73) | 0.57 |  |  | 1 | (1.4) | 6.29 (0.07-612.43) | 0.43 |  |  | 0 | (0.0) | - | - |  |  | 0.77 |  |
| DPB1 | 41:01 |  | 0 | (0.0) |  | 1 | (0.9) | 3.66 (0.04-336.73) | 0.57 |  |  | 0 | (0.0) | - | - |  |  | 1 | (2.2) | 9.71 (0.10-920.58) | 0.33 |  |  | 0.50 |  |
| DPB1 | 47:01 |  | 1 | (0.7) |  | 0 | (0.0) | 0.39 (0.004-37.87) | 0.69 |  |  | 0 | (0.0) | 0.64 (0.01-61.83) | 0.85 |  |  | 0 | (0.0) | 1.06 (0.01-96.78) | 0.98 |  |  | - |  |

AITD, autoimmune diseases; GD, Graves' disease; HD, Hashimoto's disease; OR, Odds ratio; CI, Confidence intervals.
