# Supplementary figures and images for: HLA alleles, especially amino-acid signatures of HLA-DPB1, might contribute to the molecular pathogenesis of early-onset autoimmune thyroid disease
Source: PLoS One. 2019 May 15;14(5):e0216941. doi: 10.1371/journal.pone.0216941 (PMC6519818; doi:10.1371/journal.pone.0216941)

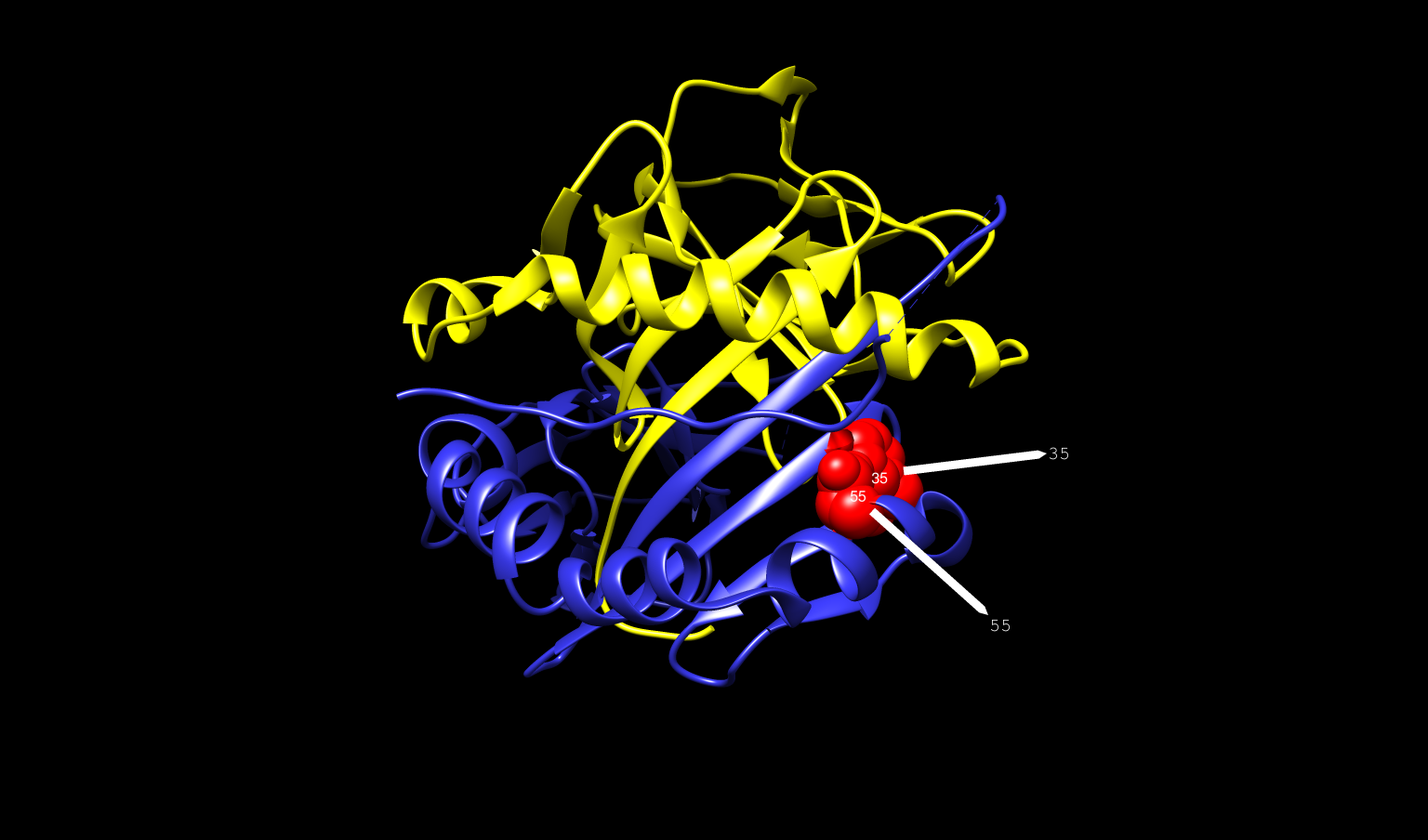

Supplement: S1 Fig — The protein structures of HLA-DPB1 is based on Protein Data Bank (PDB) (https://www.rcsb.org/) entries 3LQZ, which were prepared using UCSF (University of California, San Francisco) Chimera version 1.7. Residues at the Graves’ disease risk-associated amino acid positions are highlighted as colored spheres. (TIF) [file pone.0216941.s002.tif]
